# Prevalence and Risk Factors of Food Insecurity among Mexican University Students’ Households

**DOI:** 10.3390/nu13103426

**Published:** 2021-09-28

**Authors:** Pablo Alejandro Nava-Amante, Alejandra Betancourt-Núñez, Barbara Vizmanos, Miguel Amaury Salas-García, María Fernanda Bernal-Orozco, Elisa J. Vargas-García, Andrés Díaz-López

**Affiliations:** 1 Doctorado en Ciencias de la Nutrición Traslacional, Centro Universitario de Ciencias de la Salud (CUCS), Universidad de Guadalajara (UdeG), Guadalajara 44320, Mexico; pablo.nava@alumnos.udg.mx (P.A.N.-A.); amaury.salas@alumnos.udg.mx (M.A.S.-G.); fernanda.bernal@academicos.udg.mx (M.F.B.-O.); 2 Instituto Traslacional de Nutrigenética y Nutrigenómica, CUCS, UdeG, Guadalajara 44340, Mexico; 3 Departamento de Ciencias de la Salud, Universidad Iberoamericana León, León 37238, Mexico; elisa.vargas08@gmail.com; 4 Serra Hunter Fellow, Universitat Rovira i Virgili (URV), 43201 Reus, Spain; andres.diaz@urv.cat; 5 Nutrition and Mental Health Research Group (NUTRISAM), Rovira i Virgili University (URV), 43201 Reus, Spain; 6 Institut d’Investigació Sanitària Pere Virgili (IISPV), 43201 Reus, Spain; 7 Centro de Investigación Biomédica en Red Fisiopatología de la Obesidad y la Nutrición (CIBEROBN), Institute of Health Carlos III, 28029 Madrid, Spain

**Keywords:** college students, university students, food insecurity, food security, Mexico, Mexican

## Abstract

Household food insecurity (FI) remains a major public health challenge worldwide. Data about perceived FI and its risk factors in Mexican university students are lacking. We aimed to assess FI’s prevalence and factors affecting it among university students’ households in Mexico. This cross-sectional analysis involved 7671 university students’ households using the 2018 Mexican National of Household Income and Expenditure Survey data. Variables analyzed included sociodemographic characteristics, and the 12-item validated Mexican Scale for Food Security (*EMSA*). Multivariable logistic regression modelling was performed to identify FI risk factors. The overall household FI prevalence was 30.8%. According to FI severity, prevalence rates were 16.3% for mild-FI, 8.8% for moderate-FI, and 5.7% for severe-FI. Low socioeconomic status (OR = 2.72; 95%CI: 2.09–3.54), low education level of household’s head (OR = 2.36; 95%CI: 1.90–2.94), self-ascription to an indigenous group (OR = 1.59; 95%CI: 1.41–1.79), attending public university (OR = 1.27; 95%CI: 1.13–1.43), female-headed household (OR = 1.26; 95%CI: 1.13–1.40), having worked recently (OR = 1.19; 95%CI: 1.07–1.33), and being in second year of studies (OR = 1.17; 95%CI: 1.03–1.33), were significantly related to FI. Our results confirm that FI is highly prevalent among Mexican university students’ households and that sociodemographic factors are essential in addressing this concern. Findings highlight the need for preventive programs and policies to alleviate FI.

## 1. Introduction

Food insecurity (FI) is a multidimensional concept that exists whenever “the availability of nutritionally adequate and safe foods or the ability to acquire acceptable foods in socially acceptable ways is limited or uncertain” [1]. FI remains a significant public health challenge worldwide. According to the latest estimates, in 2019, 25.9% of the world population and 31.7% of the Latin American and Caribbean population suffered moderate or severe FI [2]. Furthermore, 22.7% of the Mexican population suffered moderate or severe FI in the 2018–2019 period [3]. However, these rates are expected to rise due to the coronavirus disease (COVID-19) pandemic [2]. Some risk factors associated with FI in the general Mexican population [4] and other contexts [5,6] are the following: woman household head, household with a family member that is disabled, a household head with no formal education, native language, having two or more children at home, living in a rural area [4], younger age [4,5,6], renting their home [5,6], being unmarried with or without children [6], and low-income household [4,6].

Child FI increases the risk of congenital disabilities, anemia, being hospitalized; cognitive, behavioral, and emotional problems, poor overall health [7] and malnutrition [8]. In addition, in adults, FI has been associated with decreased nutrient intakes, adverse mental health problems, poor health [7], diabetes [7,9], and, in women, with higher risk of overweight/obesity [10] and hypertension [9]. Moreover, in the presence of FI, people carry out behavioral modifications or coping strategies that are likely to have immediate or long-term negative consequences for health and well-being; such as the following: decrease in the quality and quantity of food (i.e., limiting the size, diversity and frequency of meals and consuming less expensive, expired, almost expired or discarded foods), emigration to seek employment, borrowing food or money, sending family members to live with other people, participating in research studies or donating blood to earn money for food, child employment, reducing health spending (i.e., spending on medicines, or performing specialized diets) or education, among other strategies [11].

Several studies have shown that university students are a group severely affected by FI. A recent review showed that the overall estimate FI prevalence in college students from United States (US) was 41% (range 10% to 70%) [12]. Another review published in 2017 documented an average FI prevalence of 42% (range: 12.5% to 84%) in college students from the US, Australia, Canada, South Africa, and Malaysia [13]. Individual, social, and environmental factors that have been positively associated with FI in college students include race/ethnic minority [13,14,15,16,17,18], young age [13,14], having children [13,17], being financially independent [13], receiving financial support [15,16,17,18], childhood history of FI [14] and, students living in rental accommodation [19].

To date, no studies have determined FI prevalence and factors affecting it among a representative sample of Mexican university students’ households [20]. Research on FI is challenging because results vary depending on the survey used, food insecurity reference period, sample selection, sample size, and characteristics of student populations [12]. Additionally, the impact of each risk factor on FI varies both between and within countries.

Therefore, the objectives of this study were to assess: (1) the prevalence and (2) the risk factors of FI among university students’ households in Mexico before the COVID-19 pandemic.

The findings will inform the need to develop public health policies aimed at those university students most at risk of FI.

## 2. Materials and Methods

### 2.1. Study Design, Data Source and Participants

A cross-sectional analysis was conducted using data from the 2018 Mexican National of Household Income and Expenditure Survey (*ENIGH, for its acronym in Spanish*). *ENIGH* is an open access database that can be downloaded from the National Institute of Statistics and Geography (*INEGI, for its acronym in Spanish*) website [21]. *ENIGH* is conducted biennially in collaboration with *INEGI* and the National Council for the Evaluation of the Social Development Policy (*CONEVAL, for its acronym in Spanish*).

Details of the survey design and methodology have been published elsewhere [22,23]. Briefly, *ENIGH* employed probabilistic, two-stage, and cluster sampling methods. The sampling unit was dwelling; the observation units were households and people, and the population under study were households and people who lived at home. All information was recorded using standardized questionnaires applied through face-to-face interviews with the household’s head, between 21 August 2018 and 28 November 2018. The information included in the *ENIGH* database is about the amount, obtention, and distribution of households’ income and expenses, and the occupational and sociodemographic characteristics of the household members. The database contains information on 74,647 households from 32 states of Mexico. The study complies with the tenets of the Declaration of Helsinki. Considering the anonymous nature of the publicly available data and that no additional contact with participants was needed, this study was not reviewed by a Research Ethics Board.

We included households with complete data of the Mexican Scale for Food Security (*EMSA, for its acronym in Spanish*) [24] and with at least one university student living at home at the time of survey application. It is possible that more than one university student lived in the household, but only one university student per household was included in the present analysis. The selection of the university student for this analysis was not intentional. There is a database with the characteristics of the participants and another database with the characteristics of the households. When merging both databases, the characteristics of a university student (irrespective of whom this was) were joined with the characteristics of their home.

### 2.2. Sociodemographic Characteristics

The following characteristics, available in the database for each university student, were selected: age, sex, marital status (single: single, separated, divorced or widower; being in a relationship: de facto or married), type of university attended (private or public), years of study (first, second or third to fifth years), receiving a scholarship (yes or no), being an indigenous speaker (yes or no), self-ascription to an indigenous group (yes, if a person considers himself/herself as indigenous, according to his/her culture), and working during the last month (yes or no).

In addition, university student’s household characteristics were included: the presence of children under 18 years of age in the household (yes or no), mother’s presence in the household (yes or no), father’s presence in the household (yes or no), household’s head sex, household’s head educational level (graduate or postgraduate; elementary, middle, or high school; no educational instruction, kindergarten, or incomplete elementary school), and population size where the household is established (≥100,000 inhabitants, urban area, large city; 2500 to 99,999 inhabitants, urban area, town; <2500 inhabitants, rural area). Socioeconomic status (high, middle-high, middle-low, or low) was also considered. The categorization was constructed by *ENIGH* personnel through a statistical analysis of 24 indicators within the household (socioeconomic, physical, and equipment features). The household was classified as nuclear (only one primary family group), one-person (home made up of a single person who is the household’s head), extended (household’s head and his/her primary family group plus other family groups or other relatives), composite (nuclear or extended household plus people unrelated to the household’s head), co-resident (household consisting of two or more people who are unrelated to the household’s head). For analytical purposes, however, the household type variable was recategorized as nuclear household and others.

### 2.3. Household Food Security Status

The degree of household food security was measured using the validated Mexican Scale *EMSA* [24]. This instrument has been used in previous studies in the Mexican population [4,25] and by *CONEVAL*, to measure FI and perceived food access [26]. The scale includes 12 questions with yes/no response options. Each item inquires if, in the last three months and due to lack of money or resources, an adult in the household ever: (1) had a low varied diet; (2) skipped breakfast, lunch, or dinner; (3) ate less than you should; (4) ran out of food; (5) felt hungry but did not eat; and (6) ate only once or stopped eating one day. Question 5 and 6 were only answered by those who answered “yes” in the first four questions. When children under 18 years of age live in the household, it additionally asks if a minor at home: (7) had a low varied diet; (8) ate less than he/she should; (9) received less amount of food at meals; (10) felt hungry but did not eat; (11) went to bed hungry, and (12) ate only once or stopped eating on a given day [24].

Based on the total number of affirmative answers on the *EMSA*, a score was obtained, ranging from 0 to 6 for households without children or 0 to 12 for households with children. According to this score, households were classified into four different categories: household food security (0 points), mild household FI (1–2 or 1–3 points for households without or with children, respectively), moderate household FI (3–4 or 4–7 points for households without or with children, respectively) and severe household FI (5–6 or 8–12 points for households without or with children, respectively) [24].

### 2.4. Statistical Analysis

Descriptive data are presented as mean and standard deviation for quantitative variables or number and percentage for qualitative variables. Differences in quantitative variables were examined using the ANOVA test, and different distribution in qualitative variables were examined using the Chi-square test. We calculated FI and food security prevalence with a 95% confidence interval (CI) in all Mexican households and each of the 32 states of Mexico.

We used univariable and multivariable logistic regression analyses to assess risk factors, including all sociodemographic characteristics, related to household FI (compared to food-secure households) and are presented as odds ratio (OR) and 95%CI. For multivariable analysis, all factors showing a *p*-value < 0.1 in the univariable analysis were included.

We excluded, in the multivariable model, the following variables: “children under 18 years of age in the household”, because it has collinearity with the dependent variable; “mother’s presence in the household” and “father’s presence in the household”, because they were highly correlated with “sex of the head of household” variable; and “indigenous speaker”, since it has collinearity with the variable “self-ascription to an indigenous group”. A choropleth map of household FI status in Mexico was generated in Microsoft Excel^®^ from Microsoft 365. Statistical analysis was performed by STATA^®^ software, version 15.0 (StataCorp, College Station, TX, USA). A *p*-value < 0.05 was established as statistically significant.

## 3. Results 

### 3.1. Participants Sociodemographic Characteristics

A total of 7671 university students and households were included in the study. Overall, the mean students’ age was 22.2 ± 5.3 years, 50.9% were female, 89.8% were not in a partner relationship, and 41.5% had worked last month. In addition, most of them were in a public or a government university (71.9%), did not receive a grant (84%), and more than a half were either in the first or second year of their studies (58.1%). Only 1.7% was an indigenous speaker, and 22.7% self-reported as belonging to an indigenous group (Table 1, total column). In terms of the composition of student household: most were middle-low or middle-high socioeconomic status (77.1%), lived in a nuclear-type family (66.7%), where no children under 18 years of age was present (74.4%), and generally the mother (78.2%) and/or the father (59.7%) were present. Most of the household’s head had elementary, middle, or high school level (71.5%) and were males (70%). Only 19.4% had a bachelor’s or postgraduate degree. Half of the households were located in large cities (≥100,000 in inhabitants) (53.9%) (Table 1, total column).

### 3.2. Household Food Insecurity Prevalence

**Figure 1:**
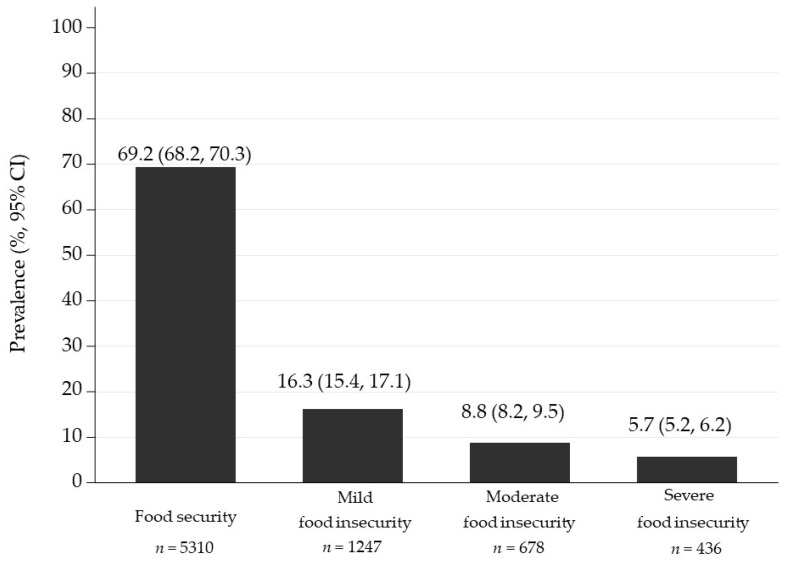
Prevalence of food security/insecurity in Mexican university students’ households, according to the Mexican Scale for Food Security (*EMSA, Spanish acronym*).

The overall FI prevalence of university student households was 30.8% (95% CI: 29.7, 31.8). The prevalence was 16.3% (95% CI: 15.4, 17.1) for mild FI, 8.8% (95% CI: 8.2, 9.5) for moderate FI, and 5.7% (95 CI: 5.2, 6.2) for severe FI (Figure 1).

**Figure 2:**
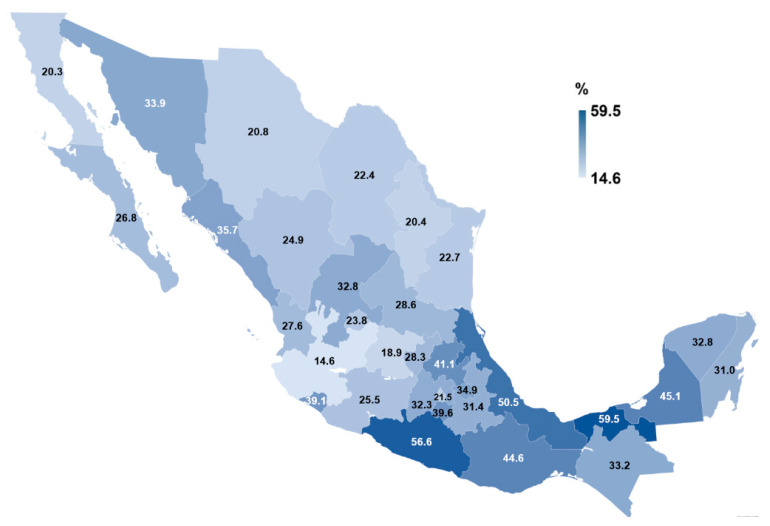
Prevalence of food insecurity in Mexican university students’ households in the 32 Mexican states according to the Mexican Scale of Food Security (*EMSA, for its Spanish* acronym). Food insecurity categories include mild, moderate, and severe food insecurity.

As shown in Figure 2, FI prevalence of households in each state of Mexico ranged from 14.6% (95%CI: 10.0, 20.3), in Jalisco, to 59.5% (95%CI: 52.3, 66.4), in Tabasco. The higher prevalence of FI was found in the southern states of: Tabasco, Guerrero (56.6%; 95%CI: 48.1, 64.9), and Veracruz (50.5%; 95%CI: 43.3, 57.7). Supplementary Table S1 describes the FI prevalence in each of Mexico’s states. Details on the total number of affirmative answers for each item on the *EMSA* are shown in Supplementary Table S2.

Table 1 shows sociodemographic students’ characteristics according to household FI categories. We observed significant differences in sociodemographic variables (*p* < 0.05), except for student sex and having a scholarship (Table 1). 

### 3.3. Association between Sociodemographic Characteristics and Household Food Insecurity 

Univariable logistic regression analysis (Table 2) revealed that attending to a public university (OR: 1.26; 95%CI: 1.13, 1.40; *p* < 0.001), being in the first year (OR: 1.22; 95%CI: 1.09, 1.37; *p* < 0.001) or second year of studies (OR: 1.24; 95%CI: 1.10, 1.41; *p* = 0.001), being an indigenous speaker (OR: 1.74; 95%CI: 1.23, 2.48; *p* = 0.002), having a self-ascription to an indigenous group (OR: 1.96; 95%CI: 1.75, 2.19; *p* < 0.001), and having worked in the last month (OR = 1.22; 95%CI: 1.10, 1.34; *p* < 0.001) were significant risk factors for household FI. Conversely, a lower risk of household FI was significantly observed with university student’s age (OR: 0.98; 95%CI: 0.97, 0.99; *p* < 0.001). 

Regarding university students’ households, we found an inverse association between socioeconomic status and FI; the higher the socioeconomic status, the lower the risk of FI (OR: 1.45; 95%CI: 1.20, 1.75 for the upper-middle class; OR: 2.37; 95%CI: 2.00, 2.82 for the lower-middle class; OR: 4.50, 95%CI: 3.63, 5.58 for the lower class; all *p* < 0.001). Similarly, living in a non-nuclear household (OR: 1.18; 95%CI: 1.06, 1.31; *p* = 0.001), maternal presence in the household (OR: 1.17; 95%CI: 1.03, 1.31; *p* = 0.012), having a female head of household (OR: 1.21; 95%CI: 1.09, 1.35; *p* < 0.001), having a head of household with low educational level (OR: 3.77; 95%CI: 3.07, 4.62; *p* < 0.001), and having their household established in a population with less than 99,999 inhabitants (OR: 1.50; 95%CI: 1.34, 1.68 for 2500 to 99,999 inhabitants; OR: 1.91; 95%CI: 1.69, 2.16 for <2500 inhabitants; all *p* < 0.001) were associated with a greater risk of household FI (Table 2). In contrast, paternal presence in the household (OR: 0.89; 95%CI: 0.81, 0.98; *p* = 0.021) was a protective factor for the risk of household FI.

Our multivariable regression model, which included variables identified with a *p*-value < 0.1 in the univariate analysis (Table 2), found that all the aforementioned factors (except being in the first year of studies, household type, and population size) remained as independent predictors of risk of household FI (all *p* < 0.05).

## 4. Discussion

A third of the Mexican university students’ households in our study live with some level of FI. The household characteristics were positively associated with FI, especially having a middle or lower socioeconomic status, a household head with a low educational level (high school or lower), and a female-headed household. Furthermore, FI was more likely in students with a self-ascription to an indigenous group, those attending a public university, those having worked last month, and those in the second year of studies. However, FI was less likely in older students. To our knowledge, this is the first study determining FI prevalence and factors affecting it among a representative sample of Mexican university students’ households.

In our study, FI prevalence was high (30.8%), similar to the mean prevalence reported in previous reviews about FI prevalence in university students in other countries (27–42%), ranging from 10% to 84% [12,13,27]. The most frequent questionnaires used to measure FI in studies on university students were the US Department of Agriculture Food Security Survey Module and the 10-item Adult Food Security Module. However, FI prevalence observed in this study was lower in comparison with the national prevalence (55%), determined with the Latin American and Caribbean Food Security Scale (*ELCSA Spanish acronym*) [3], extended version of the *EMSA*. The difference in prevalence rates may be due to the low frequency in this study of some characteristics (in less than 30% of participants) that have been positively associated with FI in studies carried out in the Mexican population [4,28,29,30] and in university students from other countries [13,15,16,18]. These characteristics (percentage in our study) are: self-ascription to a minority group (22.7%) [13,14,15,16,18,28,29,31], presence of children under 18 years of age at home (25.6%) [4,13,29], woman household’s head (30%) [4,29], households located in rural areas (<2500 inhabitants) (21%) [4,29], and household’s head with low or without education (13.1%) [4,29,30,32].

Most university students in our study lived in nuclear (66.7%) and extended (28.9%) household type. Only 2.7% had a one-person household and 0.8% a co-resident household. This living situation of university students differs from other countries. Only 35.3% of college students from Australia, lived with their parents [19], 84.4% of freshmen from US [33] and 31.4% of university students from Nigeria [34] lived on campus. This is relevant because traditional and extended households are more likely to be food secure than unitary households [4]. Besides, students living with their parents or other relatives are less likely to have FI, than students who lived alone [31]. However, there are other households and individual characteristics that could increase the FI risk in this population group.

Having low socioeconomic status was positively associated with FI. Low income is one of the main risk factors for FI. In other contexts, lower income was positively associated with FI in UK adults [5], Australian adults [35], and in US college students [36]. In the Mexican population, any income level in comparison to very low income was positively associated with food security and negatively with FI [4]. In addition, FI was more frequent in those with the lowest family income [29]. Low-income households may not be able to buy enough food to meet their needs. In 2018, 48.8% of the Mexican population (61.1 million people) were living in poverty with an insufficient income to buy the basic food basket, goods, and services; and 16.8% (21 million people) were living in extreme poverty with an insufficient income to buy the basic food basket [37,38].

Educational level of a household’s head lower than graduate or postgraduate was positively associated with FI, similar to other studies conducted on college students [32,33] and the general Mexican population [4,29,30]. Moreover, in the general population from Australia, having less than university-level education was positively associated with FI [35]. Education broadens the possibility of having a better job and a better income, allowing to acquire enough food to cover the nutritional needs of the members of the household [39]. In Mexico, the average quarterly income increased with education level. Those with postgraduate studies had the highest income, and those with an incomplete primary school had the lowest income [23,40]. Additionally, a low education level may have an impact on how household resources are managed.

Having a female-headed household was positively associated with FI, similar to studies conducted on Mexican population [4,29]. In a systematic review and meta-analysis, it was evidenced that female-headed households were 75% (95% CI: 49%, 96%) more likely to be food insecure than male-headed households [41]. This type of household is more vulnerable to deprivation situations, compared to men-headed households. Reasons behind are that: (1) they are single-parent (mother and children) or extended households, either due to separation, divorce, or widowhood [26,39]; (2) there is a greater number of members under 15 and over 65 years, that represents a greater potential economic dependence [26]. Another consideration is that (3) because of gender roles, women typically assume responsibility for domestic work and caring for other people. Therefore, women tend mainly to seek part-time jobs or reduced hours as a strategy to combine both activities. However, this implies inserting themselves into more precarious jobs, with lower remuneration and less stability [26]; (4) there is income inequality between men and women. Particularly in Mexico, although Mexican laws promote labor equality between men and women, women receive less income from their work regardless of age, education and marital status [23,26,40,42,43]. Finally, (5) feminized occupations tend to have relatively lower wages [26].

Similarly to this study, speak indigenous in general Mexican population [28,29], and being Hispanic students, black students, Asians students [14,31], mixed-race/other students [14], students of color [13], and being minority [15,16], were associated with FI. Particularly in Mexico, in 2018, compared to the non-indigenous population, the indigenous population had lower income, less access to social security (55.1% vs. 78.2%, respectively), lower housing quality and spaces (9.2% vs. 28.5%, respectively), less access to essential household services (15.7% vs. 57.5%, respectively), less access to food (19.2% vs. 31.5%, respectively), a higher percentage of educational lag (15.4% vs. 31.1%, respectively) [44], and higher percentage of poverty (39% vs. 69.5%-8.4 million people-, respectively) [23,40,44].

We observed that attending a public university was positively associated with FI. This association was not observed in a study on university students from the US [32]. In Mexico (2018–2019), there were more private schools of higher education (*n* = 3252) than public schools (*n* = 2283); however, more university students attended public schools (2,773,338 students) than private schools (1,170,206 students). Public schools obtain financing from federal, state and autonomous funds, while private schools obtain financing from private sources and subsidies [45]. Therefore, public universities are economically more accessible than private universities to students of any socioeconomic level or degree of vulnerability.

Employed students were more likely to be food insecure [36], while being a non-employed student was negatively associated with FI than students working full-time [32]. It has been evidenced that financially independent students were more likely to report FI [13,46]. Otherwise, receiving financial support from family was negatively associated with being food insecure [16]. Furthermore, students may work because they need an economic income. Nevertheless, students, to combine work and study, may work part-time and therefore, due to their student status and the work they perform, their salaries are low. In US university students, having one or more part-time jobs was positively associated with FI, with respect to not working [17,18]. Moreover, many younger students may not have the experience and skills to manage financial resources or save. A study showed that spending on other items instead of buying food was positively associated with FI in students [15]. We do not know if this happens in Mexico, but it could be that if students work, it is to meet their personal expenses (cellphone…).

Finally, in the present study, being in second year of studies was positively associated with FI compared with being in third to fifth years. Other studies also have identified academic years as predictors of university students’ FI [15,18,47]. In a longitudinal study, FI was positively associated with the end of the first semester and the end of the second semester compared with the start of the first semester [47]. In the US, students in the sophomore and junior years were more likely to experience FI than freshmen [17] or graduate students [15]. Regarding our results, students with more years of study may have more knowledge or preparation than students in the first years of university and can access a better-paying job. The former can also explain the negative association observed, in this and other studies [14,35], between age and FI.

FI is a public health problem that needs attention. Particularly in university students, FI has been positively associated with lower overall self-reported health [13,15,36], more inadequate eating behaviors (such as lower fruit and vegetable intake [13,27,48], no daily breakfast and evening meal consumption [47]), fewer days of physical activity [47,48], fewer days of enough sleep [48], poor sleep quality [33], disordered eating [33], lower grade point average (GPA) [13,15,32,33,36,49,50], lower academic progress [15], difficulty concentrating in class [13], withdrawing from class or the institution [13], lower likelihood of college graduation and obtaining a bachelor’s degree or graduate/professional degree [51], poor mental health [47,49], stress [32,33,47] and depressed mood [32,47], and higher BMI [48].

The available energy food supply in Mexico exceeds the requirements to cover the demand. The problem is that not all people have access to it [52,53]. Over the course of several years, various food policies have been implemented in Mexico [54]. In 2018, 35% of Mexican households benefited from at least one food aid program. These supports were received mainly by those living in rural locations and the southern states of Mexico [3]. In the 2012–2016 period, it was identified that food aid social programs had a positive effect on households located only in rural localities. In these households, the proportion of households with food security increased, and those with moderate and severe FI decreased [55].

On the other hand, in 2018 [56] and 2021 [57,58], the government offered financial support scholarships for university students who meet specific requirements. Additionally, public and private universities in Mexico, and non-governmental organizations (public and private), also offer financial scholarships and discounts on tuition fees for students who meet some requirements [59]. However, the main objective of this support is to promote school retention and completion of studies. To our knowledge, there are no food-type supports for university students in Mexico. Therefore, particularly for university students, it is suggested to implement financial counseling at the individual level, grant food pantries at the institutional level, and make changes at the policy level to increase financial aid [13] and create food aid programs for university students. At the national level, in addition to maintaining government programs to alleviate hunger and improving people’s nutritional status, the following are required: improvements in education, well-paying jobs, social security [38,52], availability of healthy food at affordable prices [38], and sustainable diet and production system [60].

The main strengths of this study are the large sample size, which includes participants from all states of Mexico, participants from all socioeconomic levels, and participants who live in different types of localities. It used a validated instrument to evaluate Mexicans’ household food security status (*EMSA*) [24]. Likewise, the participants in this study come from a randomly selected sample. Therefore, the FI prevalence observed probably reflects the reality of Mexican university students’ households in 2018. Moreover, these findings indicate an advance in understanding the relationship between FI and sociodemographic variables in this population group. However, the present study has some limitations. FI prevalence in university students and the general population from Mexico may have increased due to the COVID-19 pandemic. Poverty in the Mexican population increased in 2020 (55.7 million people), compared to 2018 (51.9 million people) [61]. In addition, in other contexts, due to the pandemic FI prevalence increased in US households [6], and changes in the FI prevalence are suggested in Australian adults [35], and university students [31,46]. Therefore, a current analysis of the FI prevalence in university students is required. Additionally, the cross-sectional nature of the study design limits the potential to discern causative relationships. Consequently, the results should be interpreted with caution. As the sample of this study consisted of Mexican university students’ households, the results cannot be generalized to the general population.

## 5. Conclusions

In conclusion, a high prevalence of FI was detected among Mexican university student households. One out of three Mexican households suffers from mild, moderate, or severe FI. Further characterization of sociodemographic factors underlying FI may help to identify intervention opportunities to mitigate the consequences of FI. The population of university students is affected by FI, but their study is underrated in Mexico. This analysis encourages future research focused on the consequences of FI, such as unhealthy dietary patterns, bad food choices, low academic performance, and health disorders in Mexican university students.

## Figures and Tables

**Table 1:** Sociodemographic characteristics of Mexican university students’ households according to food insecurity (FI) categories ^1^.

University Students’ SociodemographicCharacteristics	Total *n*	Food Security	Mild FI	Moderate FI	Severe FI	*p*-Value ^2^
		(*n* = 5310)	(*n* = 1247)	(*n* = 678)	(*n* = 436)	
Student age, years ^3^	22.2 ± 5.3	22.3 ± 5.5	21.8 ± 5.0	22.1 ± 5.5	21.6 ± 4.2	<0.001
Student sex ^4^						
Male	3763	2599 (69.1)	619 (16.4)	335 (8.9)	210 (5.6)	0.947
Female	3908	2711 (69.4)	628 (16.1)	343 (8.8)	226 (5.8)	
Marital status ^4^						
Single (single, separated, divorced or widower)	6887	4745 (68.9)	1133 (16.5)	602 (8.7)	407 (5.9)	0.025
In a relationship (de facto or married)	784	565 (72.1)	114 (14.5)	76 (9.7)	29 (3.7)	
Type of university attended ^4^						
Private	2158	1568 (72.7)	323 (15)	163 (7.6)	104 (4.8)	<0.001
Public	5513	3742 (67.9)	924 (16.8)	515 (9.3)	332 (6.0)	
Number of years studying ^4^						
Third to fifth year	3214	2307 (71.8)	488 (15.2)	256 (8.0)	163 (5.1)	0.007
Second year	1873	1258 (67.2)	322 (17.2)	177 (9.5)	116 (6.2)	
First year	2584	1745 (67.5)	437 (16.9)	245 (9.5)	157 (6.1)	
Has a scholarship ^4^						
No	6440	4471 (69.4)	1030 (16.0)	569 (8.8)	370 (5.8)	0.531
Yes	1231	839 (68.2)	217 (17.6)	109 (8.9)	66 (5.4)	
Indigenous speaker ^4^						
No	7542	5237 (69.4)	1223 (16.2)	660 (8.8)	422 (5.6)	0.004
Yes	129	73 (56.6)	24 (18.6)	18 (14.0)	14 (10.9)	
Self-ascription to an indigenous group ^4,5^						
No	5930	4308 (72.6)	889 (15.0)	448 (7.6)	285 (4.8)	<0.001
Yes	1741	1002 (57.6)	358 (20.6)	230 (13.2)	151 (8.7)	
Working during the last month ^4^						
No	4484	3182 (71.0)	700 (15.6)	365 (8.1)	237 (5.3)	0.001
Yes	3176	2119 (66.7)	547 (17.2)	312 (9.8)	198 (6.2)	
Socioeconomic status ^4^						
High	1035	849 (82.0)	102 (9.9)	55 (5.3)	29 (2.8)	<0.001
Middle-high	2051	1556 (75.9)	264 (12.9)	134 (6.5)	97 (4.7)	
Middle-low	3864	2542 (65.8)	686 (17.8)	390 (10.1)	246 (6.4)	
Low	721	363 (50.4)	195 (27.0)	99 (13.7)	64 (8.9)	
Household type ^4^						
Nuclear	5119	3604 (70.4)	815 (15.9)	416 (8.1)	284 (5.6)	0.004
Others ^6^	2552	1706 (66.8)	432 (16.9)	262 (10.3)	152 (6.0)	
Children under 18 years of age in the household ^4^						
No	5708	4850 (85.0)	484 (8.5)	206 (3.6)	168 (2.9)	<0.001
Yes	1963	460 (23.4)	763 (38.9)	472 (24.0)	268 (13.7)	
Mother’s presence in the household ^4^						
No	1670	1198 (71.7)	239 (14.3)	153 (9.2)	80 (4.8)	0.017
Yes	6001	4112 (68.5)	1008 (16.8)	525 (8.8)	356 (5.9)	
Father’s presence in the household ^4^						
No	3090	2093 (67.7)	493 (16.0)	293 (9.5)	211 (6.8)	0.001
Yes	4581	3217 (70.2)	754 (16.5)	385 (8.4)	225 (4.9)	
Head of the household sex ^4^						
Male	5366	3782 (70.5)	871 (16.2)	456 (8.5)	257 (4.8)	<0.001
Female	2305	1528 (66.3)	376 (16.3)	222 (9.6)	179 (7.8)	
Educational level of the head of household ^4^						
Graduate or postgraduate	1490	1245 (83.6)	143 (9.6)	64 (4.3)	38 (2.6)	<0.001
Elementary, middle, or high school	5488	3667 (66.8)	956 (17.4)	524 (9.6)	341 (6.2)	
No educational instruction, kindergarten, or incomplete elementary school	693	398 (57.4)	148 (21.4)	90 (13)	57 (8.2)	
Population size ^4^						
≥100,000 inhabitants (urban area/large city)	4060	3020 (74.4)	530 (13.1)	307 (7.6)	203 (5.0)	<0.001
2500 to 99,999 inhabitants (urban area/town)	2002	1320 (65.9)	356 (17.8)	195 (9.7)	131 (6.5)	
<2500 inhabitants (rural area)	1609	970 (60.3)	361 (22.4)	176 (10.9)	102 (6.3)	

^1^ According to the Mexican Scale for Food Security (*EMSA, Spanish acronym*). ^2^ The association between qualitative variables was analyzed with the Chi^2^ test. Comparison of age between food insecurity categories was evaluated with the one-factor ANOVA test. A *p*-value < 0.05 was established as statistically significant. ^3^ Data is presented as mean ± standard deviation. ^4^ Data is presented as frequency (percentage). ^5^ A person who considers himself/herself as indigenous, according to his/her culture. ^6^ Others: One-person household, extended household, composite household, co-resident household.

**Table 2:** Univariable and multivariable logistic regression models of sociodemographic characteristics associated with household food insecurity.

University Students’ Sociodemographic Characteristics	Household Food Insecurity ^1^			
	Univariable Model OR (95% CI)	*p*-Value	Multivariable Model ^2^ OR (95% CI)	*p*-Value
Student age	0.98 (0.97, 0.99)	<0.001	0.98 (0.97, 0.997)	0.016
Student sex				
Male	1.00 (Ref.)			
Female	0.99 (0.89, 1.09)	0.774		
Marital status				
Single (single, separated, divorced or widower)	1.00 (Ref.)		1.00 (Ref.)	
In a relationship (de facto or married)	0.86 (0.73, 1.01)	0.069	0.95 (0.79, 1.16)	0.639
Type of university attended				
Private	1.00 (Ref.)		1.00 (Ref.)	
Public	1.26 (1.13, 1.40)	<0.001	1.27 (1.13, 1.43)	<0.001
Number of years studying				
Third to fifth years	1.00 (Ref.)		1.00 (Ref.)	
Second year	1.24 (1.10, 1.41)	0.001	1.17 (1.03, 1.33)	0.017
First year	1.22 (1.09, 1.37)	<0.001	1.10 (0.97, 1.24)	0.124
Has a scholarship				
No	1.00 (Ref.)			
Yes	1.06 (0.93, 1.21)	0.377		
Indigenous speaker				
No	1.00 (Ref.)			
Yes	1.74 (1.23, 2.48)	0.002		
Self-ascription to an indigenous group ^3^				
No	1.00 (Ref.)		1.00 (Ref.)	
Yes	1.96 (1.75, 2.19)	<0.001	1.59 (1.41, 1.79)	<0.001
Working during the last month				
No	1.00 (Ref.)		1.00 (Ref.)	
Yes	1.22 (1.10 1.34)	<0.001	1.19 (1.07, 1.33)	0.001
Socioeconomic status				
High	1.00 (Ref.)		1.00 (Ref.)	
Middle-high	1.45 (1.20, 1.75)	<0.001	1.26 (1.04, 1.52)	0.020
Middle-low	2.37 (2.00, 2.82)	<0.001	1.71 (1.41, 2.07)	<0.001
Low	4.50 (3.63, 5.58)	<0.001	2.72 (2.09, 3.54)	<0.001
Household type				
Nuclear	1.00 (Ref.)		1.00 (Ref.)	
Others ^4^	1.18 (1.06, 1.31)	0.001	1.07 (0.96, 1.19)	0.250
Mother’s presence in the household				
No	1.00 (Ref.)			
Yes	1.17 (1.03, 1.31)	0.012		
Father’s presence in the household				
No	1.00 (Ref.)			
Yes	0.89 (0.81, 0.98)	0.021		
Head of household sex				
Male	1.00 (Ref.)		1.00 (Ref.)	
Female	1.21 (1.09, 1.35)	<0.001	1.26 (1.13, 1.40)	<0.001
Educational level of the head of household				
Graduate or postgraduate	1.00 (Ref.)		1.00 (Ref.)	
Elementary, middle, or high school	2.52 (2.18, 2.93)	<0.001	1.95 (1.67, 2.27)	<0.001
No educational instruction, kindergarten, or incomplete elementary school	3.77 (3.07, 4.62)	<0.001	2.36 (1.90, 2.94)	<0.001
Population size				
≥100,000 inhabitants	1.00 (Ref.)		1.00 (Ref.)	
2500 to 99,999 inhabitants	1.50 (1.34, 1.68)	<0.001	1.07 (0.94, 1.23)	0.285
<2500 inhabitants	1.91 (1.69, 2.16)	<0.001	1.05 (0.90, 1.24)	0.530

Ref., reference category; CI: confidence interval. ^1^ According to the Mexican Scale for Food Security (*EMSA for its acronym in Spanish*). The household food insecurity category includes mild, moderate, and severe food insecurity. ^2^ For multivariable logistic regression model, all factors showing a *p*-value < 0.1 in the univariable logistic regression were included. ^3^ Person who according to his/her culture considers himself/herself indigenous. ^4^ Others: One-person household, extended household, composite household, co-resident household.

## Data Availability

Publicly available datasets were analyzed in this study. This data can be found here: https://www.inegi.org.mx/programas/enigh/nc/2018/#Datos_abiertos (accessed on 5 January 2021).

## Supplementary Materials

The following are available online at https://www.mdpi.com/article/10.3390/nu13103426/s1, Table S1: Prevalence of food insecurity in Mexican university students’ households, according to state, Table S2: Perception of access to food in Mexican university students’ households.
