# Supplementary material for: Prevalence and Risk Factors of Food Insecurity among Mexican University Students’ Households
Source: Nutrients. 2021 Sep 28;13(10):3426. doi: 10.3390/nu13103426 (PMC8541269; doi:10.3390/nu13103426)
Supplement: Supplementary file 1 [file nutrients-13-03426-s001.zip › nutrients-1388618-supplementary.pdf]

Supplementary Table S1. Prevalence of food insecurity in Mexican university students' households, according to state.

States of Mexico	Total *n*	Food Security	Mild Food IInsecurity	Moderate Food Insecurity	Severe Food Insecurity
Aguascalientes	286	76.2 (70.9, 81.0) *	14.3 (10.5, 18.9)	7.0 (4.3, 10.6)	2.4 (1.0, 5.0)
Baja California	320	79.7 (74.9, 84.0)	11.3 (8.0, 15.2)	4.7 (2.6, 7.6)	4.4 (2.4, 7.2)
Baja California Sur	220	73.2 (66.8, 78.9)	12.7 (8.6, 17.9)	8.6 (5.3, 13.2)	5.5 (2.8, 9.3)
Campeche	213	54.9 (48.0, 61.7)	19.2 (14.2, 25.2)	13.1 (8.9, 18.4)	12.7 (8.5, 17.9)
Coahuila	330	77.6 (72.7, 82.0)	10.9 (7.8, 14.8)	8.5 (5.7, 12.0)	3.0 (1.5, 5.5)
Colima	215	60.9 (54.1, 67.5)	21.4 (16.1, 27.5)	11.6 (7.7, 16.7)	6.0 (3.3, 10.1)
Chiapas	220	66.8 (60.2, 73.0)	20.5 (15.3, 26.4)	10.9 (7.1, 15.8)	1.8 (0.5, 4.6)
Chihuahua	379	79.2 (74.7, 83.1)	9.8 (7.0, 13.2)	5.5 (3.5, 8.3)	5.5 (3.5, 8.3)
Ciudad de México	311	78.5 (73.5, 82.9)	13.2 (9.6, 17.5)	5.5 (3.2, 8.6)	2.9 (1.3, 5.4)
Durango	233	75.1 (69.0, 80.5)	13.3 (9.2, 18.4)	5.6 (3.0, 9.4)	6.0 (3.3, 9.9)
Guanajuato	212	81.1 (75.2, 86.2)	10.8 (7.0, 15.8)	6.1 (3.3, 10.3)	1.9 (0.5, 4.8)
Guerrero	143	43.4 (35.1, 51.9)	27.3 (20.2, 35.3)	20.3 (14.0, 27.8)	9.1 (4.9, 15.0)
Hidalgo	174	58.6 (50.9, 66.0)	27.0 (20.6, 34.3)	8.0 (4.5, 13.1)	6.3 (3.2, 11.0)
Jalisco	199	85.4 (79.7, 90.0)	9.0 (5.4, 13.9)	3.0 (1.1, 6.4)	2.5 (0.8, 5.8)
Estado de México	331	67.7 (62.3, 72.7)	16.9 (13.0, 21.4)	10.3 (7.2, 14.1)	5.1 (3.0, 8.1)
Michoacán	161	74.5 (67.1, 81.1)	11.2 (6.8, 17.1)	8.7 (4.8, 14.2)	5.6 (2.6, 10.3)
Morelia	212	60.4 (53.5, 67.0)	23.1 (17.6, 29.4)	9.9 (6.2, 14.7)	6.6 (3.7, 10.8)
Nayarit	185	72.4 (65.4, 78.7)	16.8 (11.7, 22.9)	6.5 (3.4, 11.1)	4.3 (1.9, 8.3)
Nuevo León	285	79.6 (74.5, 84.2)	9.5 (6.3, 13.5)	5.6 (3.2, 9.0)	5.3 (3.0, 8.5)
Oaxaca	148	55.4 (47.0, 63.6)	25.7 (18.9, 33.5)	10.1 (5.8, 16.2)	8.8 (4.8, 14.6)
Puebla	185	68.6 (61.4, 75.3)	17.3 (12.1, 23.5)	7.0 (3.8, 11.7)	7.0 (3.8, 11.7)
Querétaro	307	71.7 (66.3, 76.6)	16.3 (12.3, 20.9)	7.5 (4.8, 11.0)	4.6 (2.5, 7.5)
Quintana Roo	168	69.0 (61.5, 75.9)	18.5 (12.9, 25.2)	8.9 (5.1, 14.3)	3.6 (1.3, 7.6)
San Luis Potosí	196	71.4 (64.6, 77.6)	13.8 (9.3, 19.4)	7.1 (4.0, 11.7)	7.7 (4.3, 12.3)
Sinaloa	507	64.3 (60.0, 68.5)	19.1 (15.8, 22.8)	10.8 (8.3, 13.9)	5.7 (3.9, 8.1)
Sonora	236	66.1 (59.7, 72.1)	17.4 (12.8, 22.8)	8.9 (5.6, 13.3)	7.6 (4.6, 11.8)
Tabasco	200	40.5 (33.6, 47.7)	19.0 (13.8, 25.1)	21.0 (15.6, 27.3)	19.5 (14.2, 25.7)
Tamaulipas	229	77.3 (71.3, 82.6)	11.8 (7.9, 16.7)	6.1 (3.4, 10.0)	4.8 (2.4, 8.4)
Tlaxcala	249	65.1 (58.8, 71.0)	19.3 (14.6, 24.7)	10.4 (6.9, 14.9)	5.2 (2.8, 8.8)
Veracruz	196	49.5 (42.3, 56.7)	28.1 (21.9, 34.9)	14.3 (9.7, 20.0)	8.2 (4.7, 12.9)
Yucatán	232	67.2 (60.8, 73.2)	16.8 (12.2, 22.3)	11.6 (7.8, 16.5)	4.3 (2.1, 7.8)
Zacatecas	189	67.2 (60.0, 73.8)	18.0 (12.8, 24.2)	8.5 (4.9, 13.4)	6.3 (3.3, 10.8)

*Percentage (95% CI).

Supplementary Table S2. Perception of access to food in Mexican university students' households.

**Questions from Mexican Scale for Food Security (*EMSA for its Acronym in Spanish*)**	
	Yes
In the last three months due to the lack of money or resources, have **you or any adult** at home…?	
1) Had a low varied diet?	2012 (26.2) [1]
2) Skipped breakfast, lunch, or dinner?	778 (10.1) [1]
3) Eaten less than you should?	1464 (19.1) [1]
4) Ran out of food?	649 (8.5) [1]
5) Felt hungry but did not eat?	840 (27.1) [2]
6) Eaten only once or stopped eating one day?	551 (17.8) [2]
In the last three months and due to lack of money or resources, has anyone at home **under the age of 18** …?	
7) Had a low varied diet?	828 (42.2) [3]
8) Eaten less than you should?	552 (28.1) [3]
9) Received less food during meals?	535 (27.3) [3]
10) Felt hungry but did not eat?	230 (11.7) [3]
11) Went to bed hungry?	221 (11.3) [3]
12) Eaten only once or stopped eating one day?	149 (7.6) [3]

Question 5 and 6 were only answered by those who answered "yes" in the first questions. [1] n = 7671; [2] n = 3103; [3] n = 1963.
